# Draft genome sequence of *Kocuria indica* DP-K7, a methyl red degrading actinobacterium

**DOI:** 10.1007/s13205-020-2136-3

**Published:** 2020-03-23

**Authors:** Selvapravin Kumaran, Anna Christina R. Ngo, Fabian Peter Josef Schultes, Dirk Tischler

**Affiliations:** Microbial Biotechnology, Ruhr-Universität Bochum, Universitätsstr. 150, 44780 Bochum, Germany

**Keywords:** Azo dye, Azoreductase, Dye degradation, Biodegradation, Soil bacteria

## Abstract

In the present study, we report the draft genome of soil isolate DP-K7 that has the potential to degrade methyl red. The 16S rRNA gene sequencing and whole-genome analysis exposed that the bacterial strain DP-K7 belongs to the species *Kocuria indica*. The genome annotation of the strain DP-K7 through the bioinformatics tool “Prokka” showed that the genome contains 3,010,594 bp with 69.01% GC content. The genome comprises 57 contigs including 2 rRNA genes, 47 tRNA genes, and 2754 CDS. The plate and broth assay showed that the strain DP-K7 has the potential to utilize methyl red as the sole carbon source for growth. Indeed, the RP-HPLC analysis proved that the strain DP-K7 is capable of degrading methyl red. The genome BLAST against a characterized azoreductase (AzoB—*Xenophilus azovorans* KF46F) revealed the presence of two azoreductase-like genes (*azoKi-1* and *azoKi-2*). The phylogenetic analysis of the primary amino acid sequence of characterized azoreductases suggested that AzoKi-1 and AzoKi-2 belong to members of the clade IV azoreductase, which are flavin-independent. The multiple sequence alignment of AzoKi-1 and AzoKi-2 with flavin-independent azoreductases showed the presence of NAD(P)H binding like motif (GxxGxxG). In addition, other genes coding for dye degrading enzymes (SodC, SodA, KatA, KatE, and DyP2) were also found in the genome supporting that the strain *K. indica* DP-K7 is a potential azo dye degrader.

**Electronic supplementary material:**

The online version of this article (10.1007/s13205-020-2136-3) contains supplementary material, which is available to authorized users.

## Introduction

The dyes featured with (R_1_–N = N–R_2_) are characterized as azo dyes that are widely used in various industrial products including food, paper, pharmaceuticals, and textiles (Ayed et al. 2011). Discharge of untreated industrial dyes is the source of environmental contamination (Stolz 2001). The ineffectiveness in wastewater treatment using physical and chemical methods paved the way for biological treatment of azo dyes (Solís et al. 2012). Microbial degradation of azo dyes is either aerobic or anaerobic or by a combined process (McMullan et al. 2001). Degradation of azo dye by various fungi and bacteria were reported previously by Suzuki et al. (2001), Blümel et al. (2002) and Pricelius et al. (2007). Several Gram-positive bacteria like *Bacillus cereus* and *Bacillus subtilis* were reported earlier for azo dye degradation. Gram-negative bacteria like *Proteus mirabilis, Pseudomonas luteola*, and other *Pseudomonas* spp. were also reported to degrade azo dyes under anoxic conditions (Chen et al. 1999; Chang et al. 2001; Kalyani et al. 2009; Yu et al. 2001). Besides members of the phylum Firmicutes and Proteobacteria, *Rhodococcus opacus* strain 1CP, an actinobacterium, was studied for azo dye degradation (Qi et al. 2016). Enzymatic degradation of such azo dyes was also studied as an alternative method. Based on the efficiency in enzyme production and degradation, enzymes like laccase and azoreductase were chosen for dye degradation studies (Sarkar et al. 2017). Azoreductase, a unique participant in azo dye degradation (Misal and Gawai 2018), can be classified into two groups namely, flavin-dependent and flavin-independent azoreductase which uses NADH/NADPH as an electron donor. Several bacterial species namely *Bacillus badius*, *B. cereus*, *Enterococcus faecalis* ATCC 27274, *Gracilibacillus* sp. GTY, *Pseudomonas aeruginosa*, *P. luteola*, *E. coli*, *Sphingomonas* sp. BN6, and *Staphylococcus aureus* ATCC 25923 were reported for the presence and activity of azoreductase (Chengalroyen and Dabbs 2013). So far, a few flavin-independent azoreductases have been studied such as enzymes from *Pigmentiphaga kullae* K24, *Xenophilus azovorans* KF46F, *Citrobacter* sp. KCTC 18061P, and *Klebsiella oxytoca* GS-4-08 (Blümel and Stolz 2003; Chen et al. 2010; Blümel et al. 2002; Gao et al. 2015; Hua and Yu 2019, respectively).

Based on the unique 16S rRNA gene sequences, three species of genus *Micrococcus* (*M. roseus, M. varians,* and *M. kristinae*) were grouped into a unique clade which was later coined as genus *Kocuria*, to honor the Slovakian microbiologist Miroslav Kocur (Stackebrandt et al. 1995). The genus *Kocuria* belongs to the family *Micrococcaceae* under the class actinobacteria. The species of the genus *Kocuria* possess coccoid cells, Gram-staining positive, non-motile, aerobic cells with DNA G + C content 66–75 (mol%) (Stackebrandt et al. 1995). At the time of writing, the genus *Kocuria* comprised 27 validly published species (https://www.bacterio.net/kocuria.html) based on the LPSN website (Parte 2014). The stain DP-K7 was recovered from non-rhizosphere wet soil from the forest area in Bochum, Germany. The study strain DP-K7, which is phylogenetically related to *Kocuria indica*, was initially isolated from Chorao Island, India (Dastager et al. 2014). A total of 6 whole-genome sequences are currently available in GenBank; however only the strain CE7, isolated from human skin, has a complete genome report (Lee et al. 2019). In the present study, a draft genome of *K. indica* strain DP-K7 was described in relation to azo dye (methyl red) degradation. An insight into the genome of this environmentally significant strain allows to improve our understanding of the genetic features attributed to the biodegradation of azo dyes.

## Materials and methods

### Isolation of bacterial strain DP-K7

The strain DP-K7 was isolated from the non-rhizosphere subsurface soil. The soil was collected at the depth of 30 cm from the surface at a forest in Querenburg, Bochum, Germany (51.450861 N, 7.254250 E). The isolation was carried out by the spread plate technique using Soil Extract Agar Medium (SE agar medium; SEM) as described earlier (Nguyen et al. 2018) with 200 µM methyl red (Methyl red; MR) as the sole carbon source. To ascertain the phylogeny of the strain, the 16S rRNA gene was amplified using colony PCR technique with the specific bacterial primers 27F and 1522R (Frank et al. 2008) and sequenced. The partial16S rRNA gene was submitted to NCBI GenBank under the accession number (MN689807). The evolutionary relationship was determined by calculating pairwise 16S rRNA gene similarity with nearest type strains available in EzBioCloud Database (https://eztaxon-e.ezbiocloud.net/) (Yoon et al. 2017). A pairwise sequence alignment was done with CLUSTAL W tool; evolutionary distance was determined using Kimura 2-parameter model clustering with neighbor-joining, maximum likelihood, maximum-parsimony methods of Saitou and Nei (1987), Felsenstein (1981) and Fitch (1971), respectively; and the phylogenetic tree was constructed using neighbor-joining method with MEGA 7.0 (Kumar et al. 2016).

### Characterization of azo dye degrading attributes by the strain DP-K7

Azo dye degradation potential was tested with plate and broth assays. Initially, the strain DP-K7 was grown on the SEM agar plate (Nguyen et al. 2018) supplemented with 200 µM MR (azo dye; CAS: 493-52-7) as the sole carbon source and tested for dye degradation. The broth assay was conducted subsequently, for which 10 mL SEM broth (0.2% w/v glucose) was prepared and inoculated with a single isolated colony of strain DP-K7, incubated overnight at 30 °C, 120 rpm. The pre-culture was added to 30 mL of SEM liquid broth supplemented with 200 µM MR as the sole carbon source by normalizing the OD_600_ of pre-culture to 0.1. The experiment was conducted in triplicate and the broth cultures were incubated at 30 °C, 120 rpm.

The growth curve of the strain DP-K7 was depicted as described in Qi et al. (2016), by measuring the cell turbidity at OD_600_ at regular time intervals. For evaluating the decolorization, 1 mL of culture broth were collected, spin down at 17,000×*g* for 10 min, and 500 µL of cell-free supernatant was diluted with 50 mM phosphate buffer at 1:1 ratio and measured at 430 nm (which is the absorption wavelength of MR, extinction coefficient: *ε* = 23,360 M^−1^ cm^−1^ at pH 7.0) (Chen et al. 2005) with spectrophotometer using Spectramanager V-750 via fixed wavelength measurement. The MR consumption was calculated using the formula, consumption (%) = [(initial absorbance − observed absorbance)/initial absorbance] × 100. The rest of the 500 µL supernatant was transferred to a new Eppendorf tube and stored at − 20 °C for RP-HPLC analysis.

The biodegradation of MR by the strain DP-K7 was evaluated by RP-HPLC analysis. The cell-free supernatant was diluted with HPLC-grade methanol at a 1:1 ratio. Substrate and product elution were measured by UV–vis profile at 490 nm. Respectively, the samples were analyzed with RP-HPLC using the Knauer Eurospher 100-5 C18 column, 125 × 4 mm with a gradient of 40–95% methanol and H_2_O with 0.1% TFA (trifluoroacetic acid) and a flow rate of 0.7 mL/min. Proper standards of MR and 2-aminobenzoic acid (one of the MR degradation products) were treated similarly and measured for comparison. The biodegradation of MR was evaluated by the area of eluted MR at retention time 9.51 min. The area was translated into concentration by the formula, MR concentration = [(observed area of sample/standard area of blank) × concentration of MR used (200 µM)].

### Genome sequencing, assembly, and annotation

For genomic DNA isolation, the strain DP-K7 was grown in 100 mL nutrient broth (Nutrient broth; NB) overnight at 30 °C, 120 rpm. The DNA isolation was carried out as described by Marmur (1961) and the quality of the DNA was ascertained with 1% agarose gel. The genome was sequenced using Illumina chemistry on a Genome sequencer Illumina Hiseq 2500 platform with an average read length of 150 (Eurofins Genomics, Ebersberg, Germany) (Sollars et al. 2017). A paired-end read sequencing was applied. The raw genome reads were uploaded to the web-based genome analyzer tool Galaxy-Europe (https://usegalaxy.eu/). The quality of the reads was initially analyzed using the tool “FastQC”, trimmed with “Trim Galore” and the trimmed sequences were assembled using the tool “Unicycler” (Wick et al. 2017). Therefore, the contigs shorter than 1000 bp were excluded. Assembled genome quality was verified using “Quast” (Gurevich et al. 2013) and finally, the assembled genome was annotated using the tool “Prokka” (Seemann 2014). The genome coverage of annotated genes was obtained using RAST (version 2.0) (Aziz et al. 2008). The gene clusters for secondary metabolites were analyzed with antiSMASH version 5.0.0 (Blin et al. 2019). The genomic islands were predicted via IslandViewer 4 (Bertelli et al. 2017). A general overview of the various biological features coded as subsystem in the annotated genome of DK-K7 was analyzed using RAST. In addition to “Prokka”-based annotation, the annotated genome was analyzed with the PATRIC genome analysis server (https://www.patricbrc.org/) (Wattam et al. 2017), mainly to identify the genome and protein features along with antimicrobial resistance genes. The circular genome map was also constructed with the same PATRIC tool. The genome-based phylogeny was constructed using platform “Type (Strain) Genome Server” (TYGS) (Meier-Kolthoff and Göker 2019).

To identify the azoreductase like enzyme coding genes, the BLAST search was performed with annotated genome against well-studied azoreductases such as (AzoB) from *X. azovorans* KF46F (Bürger and Stolz 2010) and AzoRo from *R. opacus* 1CP (Qi et al. 2016). This analysis was done at amino acid sequence level. A phylogenetic tree was constructed based on the primary amino acid sequence reported earlier (Suzuki 2019) along with the best BLAST hits from the genome of the strain DP-K7. All the amino acid sequences were retrieved from public domain NCBI (https://www.ncbi.nlm.nih.gov/protein). Pairwise and multiple sequence alignment was conducted using the CLUSTAL W program and phylogeny was reconstructed using minimum-evolution (ME) method with MEGA version 7.0 (Kumar et al. 2016). Clustal O, an online-based sequence alignment tool from EMBL-EBI, was used to detect the presence of conserved NAD(P)H binding like motif in azoreductase (Madeira et al. 2019).

## Results and discussion

The isolate recovered from the soil was small, round and pink-pigmented colony when grown in NB agar plate. The strain showed optimum growth at 30 °C, pH 7.0 with a doubling time of 40 h in SEM.

**Fig. 1:**
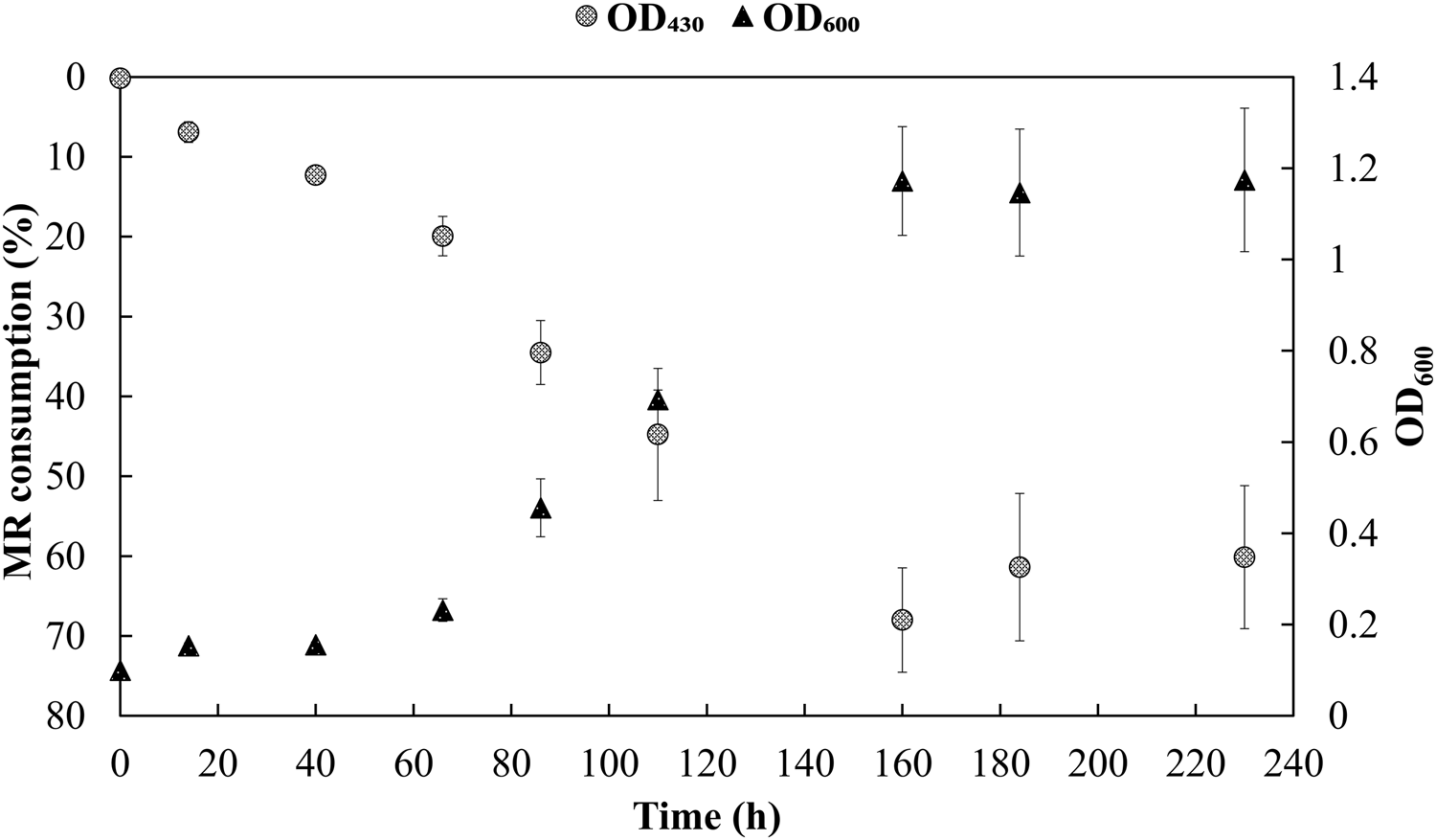
Spectrophotometric analysis for growth pattern and MR degradation by the strain *Kocuria indica* DP-K7. The growth was tracked by measuring the culture turbidity at OD_600_. MR consumption was tracked by measuring cell-free supernatant at OD_430_ and translated into relative percentage gradient. Experiments were conducted in triplicates and values were measured for every samples. The time intervals were given in *X*-axis while MR consumption and OD_600_ of strain DP-K7 were given in primary and secondary *Y*-axis, respectively

**Fig. 2:**
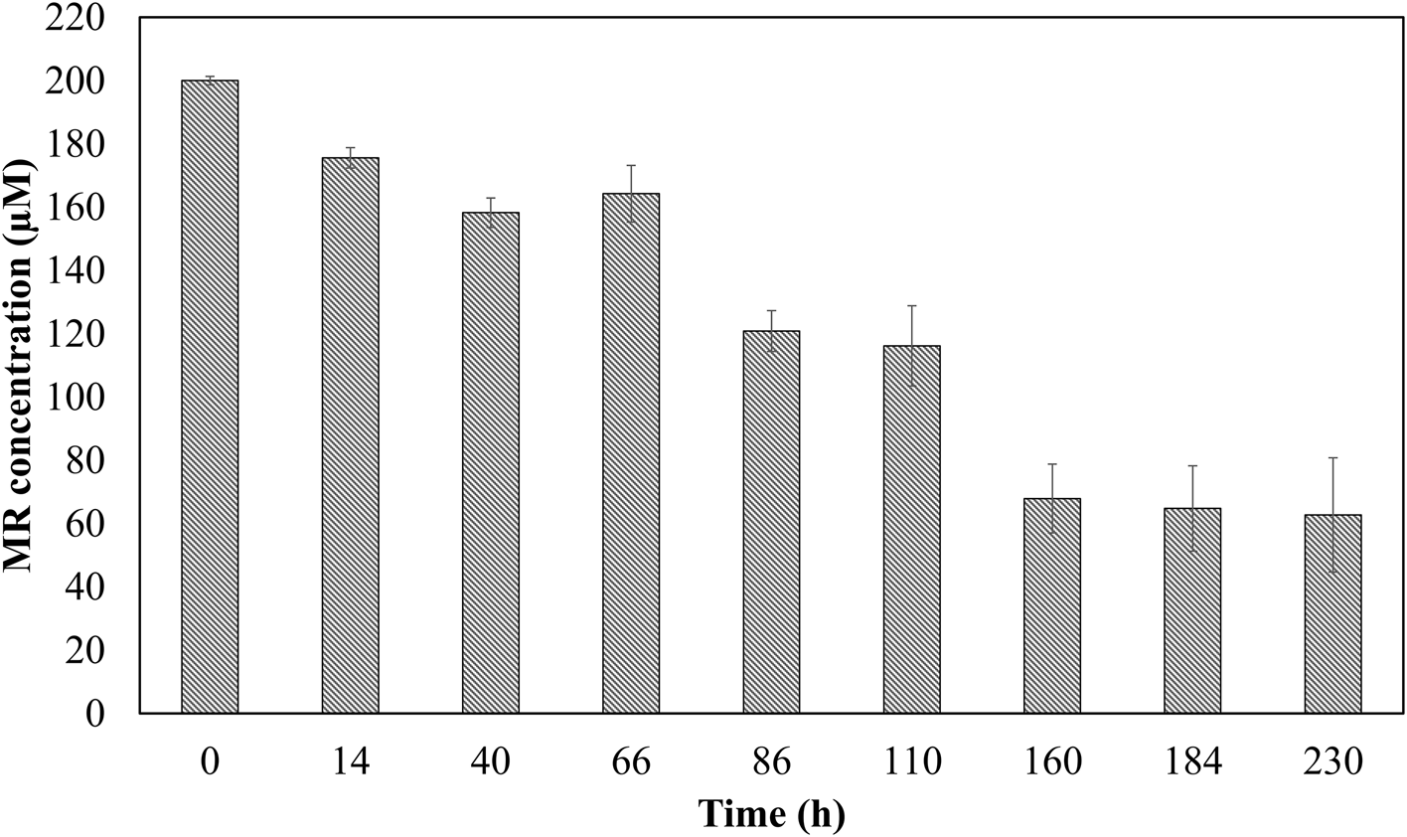
RP-HPLC analysis for biodegradation of MR by the strain *Kocuria indica* DP-K7. Cell-free supernatant was measured at UV–Vis 490 nm. Experiments were conducted in triplicates and values were measured for every samples. Vertical bar indicates the concentration of MR translated from area of peaks. *X*-axis indicates the time intervals of the analyzed samples and *Y*-axis MR concentration (µM)

To detect the biodegradation of MR, the growth of the strain DP-K7 was observed in SEM agar and liquid broth supplemented with 200 µM MR as the sole carbon source. Since the azo dye-degrading enzymes are produced in the late log phase or early stationary phase, understanding the growth pattern of the strain is mandatory. The spectrophotometric analysis (Fig. 1) showed the maximum consumption of 68% MR (an average value of triplicate readings) after 160 h of incubation. In the growth curve, cells recorded the maximum optical density of 1.2 in 160 h (Fig. 1). This shows the growth pattern and the dye consumption of the strain correlates well, as the reduction of MR ceases when the cells enter into stationary phase after 160 h of incubation. The prolonged growth pattern was due to the absence of an additional carbon sources other than MR. Analysis of the cell-free supernatant from the culture samples via RP-HPLC ensured the reduction in MR peak (Retention time—9.51 min) when compared with proper MR standard (Fig. 2). The reduction in MR peak confirms the biodegradation of MR. However, no peaks were observed at the retention time of ABA standard (degradation product of MR) and at any other time point. This suggests that the strain either has a different azo dye degradation pathway or possesses enzymes that utilize ABA for which the product could not be detected via HPLC. Indeed, the gene coding for ABA degradation, anthranilate 1,2-dioxygenase (*antA*) was present in the genome of DP-K7, sturdily suggesting that ABA might also be utilized by the strain. These results clearly reflect that the strain DP-K7 is a potential MR degrader.

**Scheme 1:**
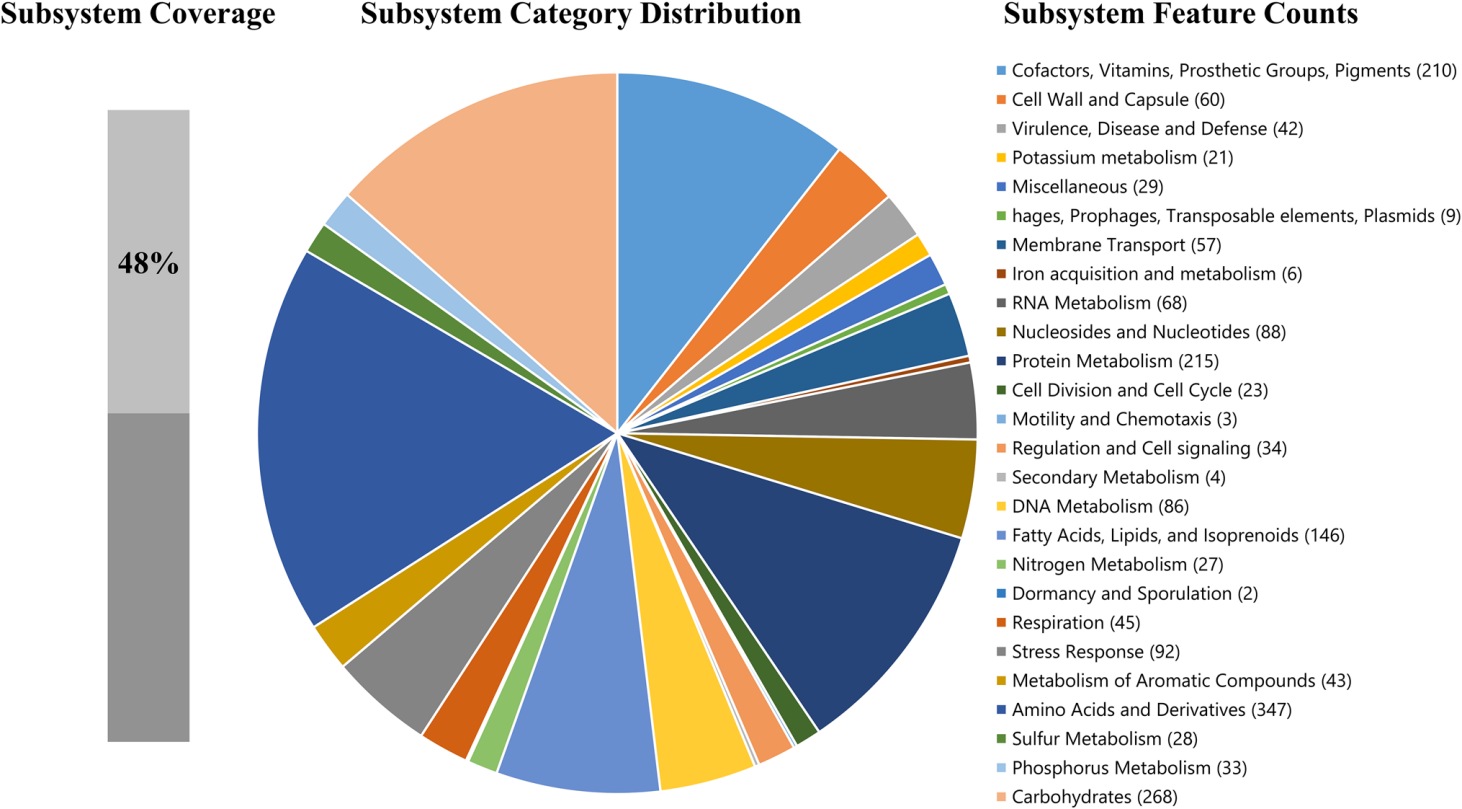
Subsystem distribution on biological functions of *Kocuria indica* DP-K7 based on Rast genome analysis server. The percentage distribution of genes was shown in different color and its corresponding number of gene features were given in numerical within parentheses

**Fig. 3:**
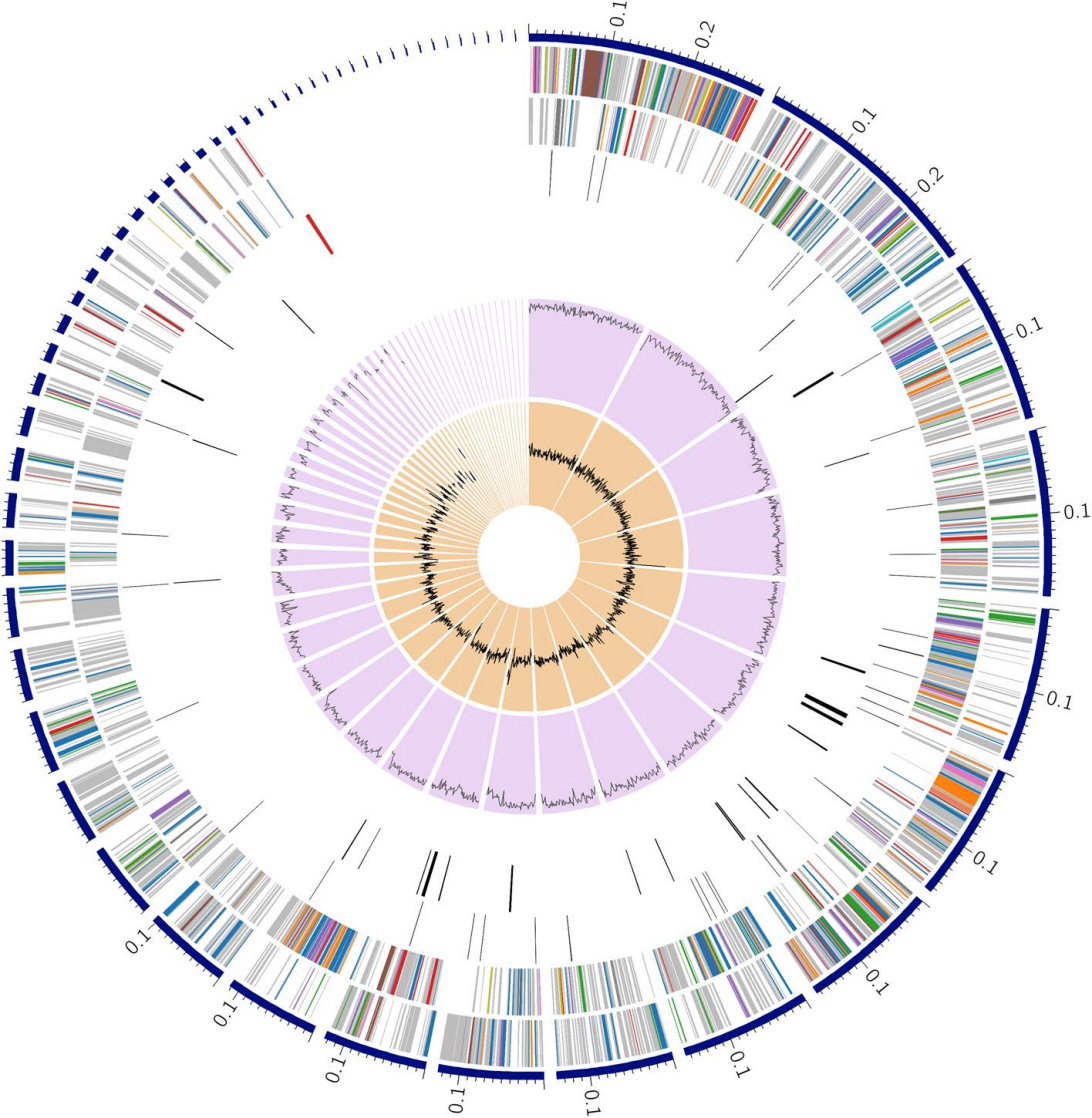
Circular genome map of *Kocuria indica* DP-K7. The overview of genome map of *Kocuria indica* DP-K7 based on 57 contigs generated via PATRIC. From outer to inner ring—contigs (scale—x1Mbp), CDS on the forward strand, CDS on the reverse strand, RNA genes, CDS with homology to known antimicrobial resistance genes, CDS with homology to know virulence factors, GC content and GC skew

The genome sequencing of the strain DP-K7 yielded 2,605,470,000 bases with 8,684,900 reads. The unicycler assembly resulted in 57 contigs ≥ 1000 bp. The quality values of N50 and N75 were 156,175 and 89,803 with L50 and L75 were 8 and 14, respectively. The 57 contigs cover a total length of 3,010,594 bp with the largest contig of 287,453 and G + C % content of 69.01%. In the genome, a total of six gene clusters for the production of secondary metabolites were predicted using antiSMASH (Blin et al. 2019) and nine genomic islands were identified in the genome by island prediction (Bertelli et al. 2017). With respect to the antiSMASH prediction, the similarity of potential secondary metabolite-related gene clusters was very low and thus the results listed here have to be taken carefully: betalactone (7% similarity with microansamycin gene cluster), T3PKS, siderophore, NRPS-like gene, terpene (25% similarity with carotenoid gene cluster), and bacteriocin. With the RAST subsystem analysis, in the annotated genome of DP-K7, 48% genes code for the subsystem that entitles the biological functions of the organism (Scheme 1). The remaining 52% could not be assigned to functional subsystems but still could code for those which need to be verified on a single case-by-case study. Analysis of ‘Prokka’ annotated genome via online-based bioinformatics tool, PATRIC, produced circular genome map (Fig. 3) and resulted in identification of gene features with two ribosomal RNA (rRNA) and 47 transfer RNA (tRNA) including protein features with 2754 protein coding sequences (CDS) out of which 757 are hypothetical proteins and the remaining with functional assignments. The 1997 functional proteins comprise 598 proteins mapping to the KEGG pathway, 669 proteins with Gene Ontology (GO) assignment and 765 proteins with Enzyme Commission (EC) number. The genome of strain DP-K7 contains 2451 proteins with genus-specific family (PLfam) assignments and 2528 proteins with cross-genus family (PGfam) assignments. In addition, many antimicrobial resistance genes such as *alr, ddl, dxr, efG, efTuEF-Tu, folA, dfr, folP, gyrA, gyrB, Iso- tRNA, kasA, murA, rho, rpoB, rpoC, S10p, S12p, erm(X), fabG, fabL-like, htdX, gidB, lpqB, mtrA, mtrB, GgdpD, *and *pgsA* were detected by k-mer-based AMR gene detection method via “PATRIC”.

**Fig. 4:**
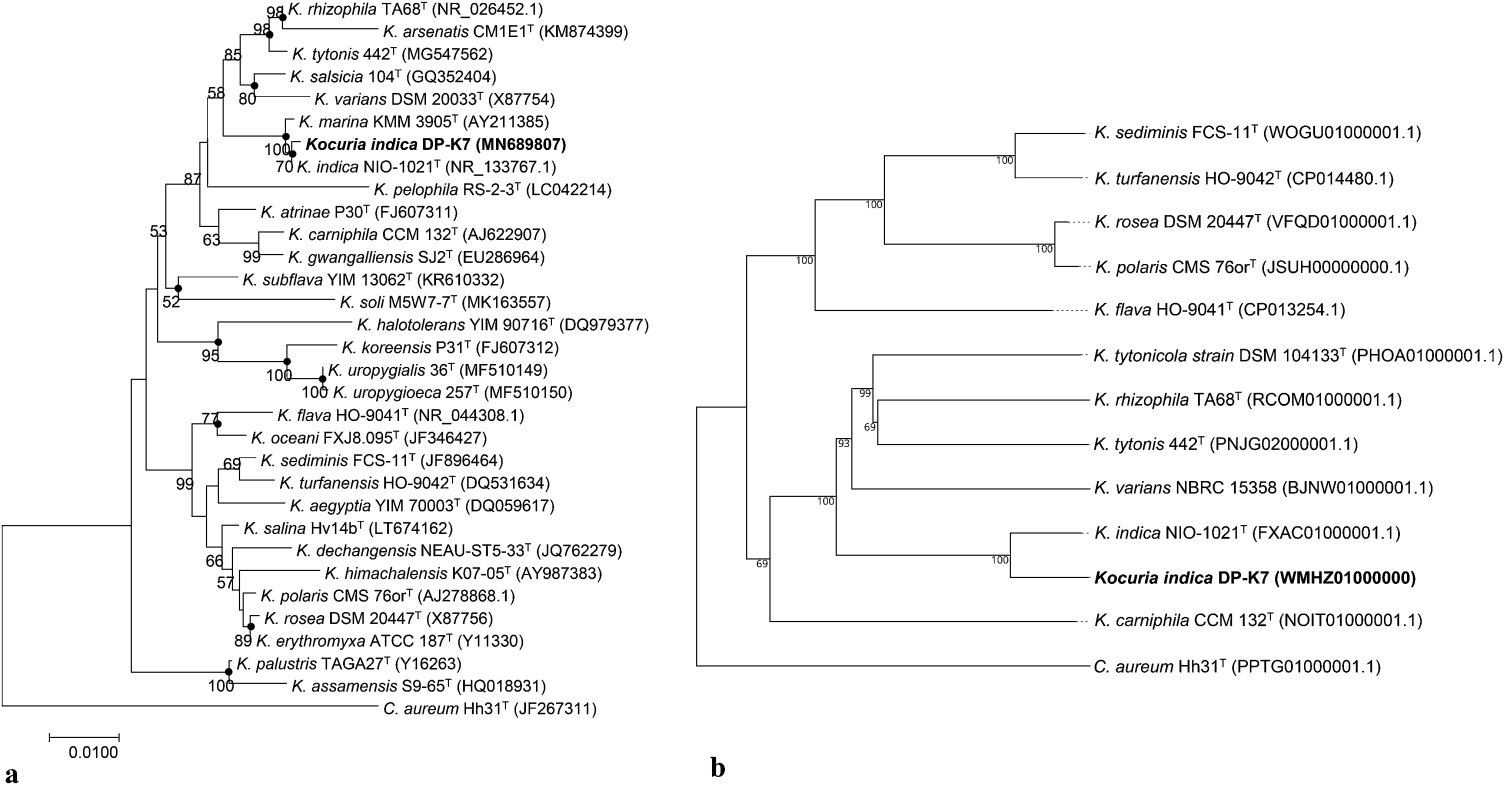
Phylogeny of *Kocuria indica* DP-K7. **a** Neighbor-joining tree based on 16S rRNA gene sequence data, depicting the phylogenetic position of the strain DP-K7 among the recognized type strains of the genus *Kocuria. Cryobacterium aureum* Hh31^T^ was used as outgroup. Black filled circles at nodes indicate generic branches that were also recovered by maximum-likelihood and maximum-parsimony algorithms. Numbers at nodes indicate the percentage of bootstrap support based on 1000 bootstrap replicates under neighbor-joining analysis. Bootstrap values below 50% were not shown. Bar 0.01% substitution per site. **b** The Minimum evolution tree based on genome sequence of recognized strains from the genus *Kocuria* was constructed via TYGS. *Cryobacterium aureum* Hh31^T^ was used as outgroup. The accessions for respective genome were provided within brackets. The tree was constructed with bootstrap support based on 100 pseudo-bootstrap replicates. The strain used in this study was provided in bold

The results of phylogenetic analysis based on the 16S rRNA gene sequence exposed that the strain DP-K7 belongs to the clade of closely related species *K. indica* NIO-1021^T^ and *Kocuria marina* KMM 3905^T^ with 99.93% and 99.71% sequence similarity, respectively (with 1 and 4 nucleotide difference). Few other closely related species are *Kocuria salsicia* 104^T^ (98.30%), *Kocuria rhizophila* TA68^T^ (98.08%), and *Kocuria varians* DSM 20033^T^ (97.80%). In all three phylogenetic treeing methods, strain DP-K7 showed similar tree topology by cladding with *K. indica* (only tree constructed with neighbor-joining method shown herein) (Fig. 4a) and the genome-based phylogenetic tree (Fig. 4b) constructed via TYGS placed the strain DP-K7 within the clade of *K. indica* NIO-1021*.* Based on the genome and 16S rRNA phylogenetic trees, it is concluded that the strain DP-K7 belongs to the species *Kocuria indica.*

### In silico analysis of azoreductase coding genes

**Fig. 5:**
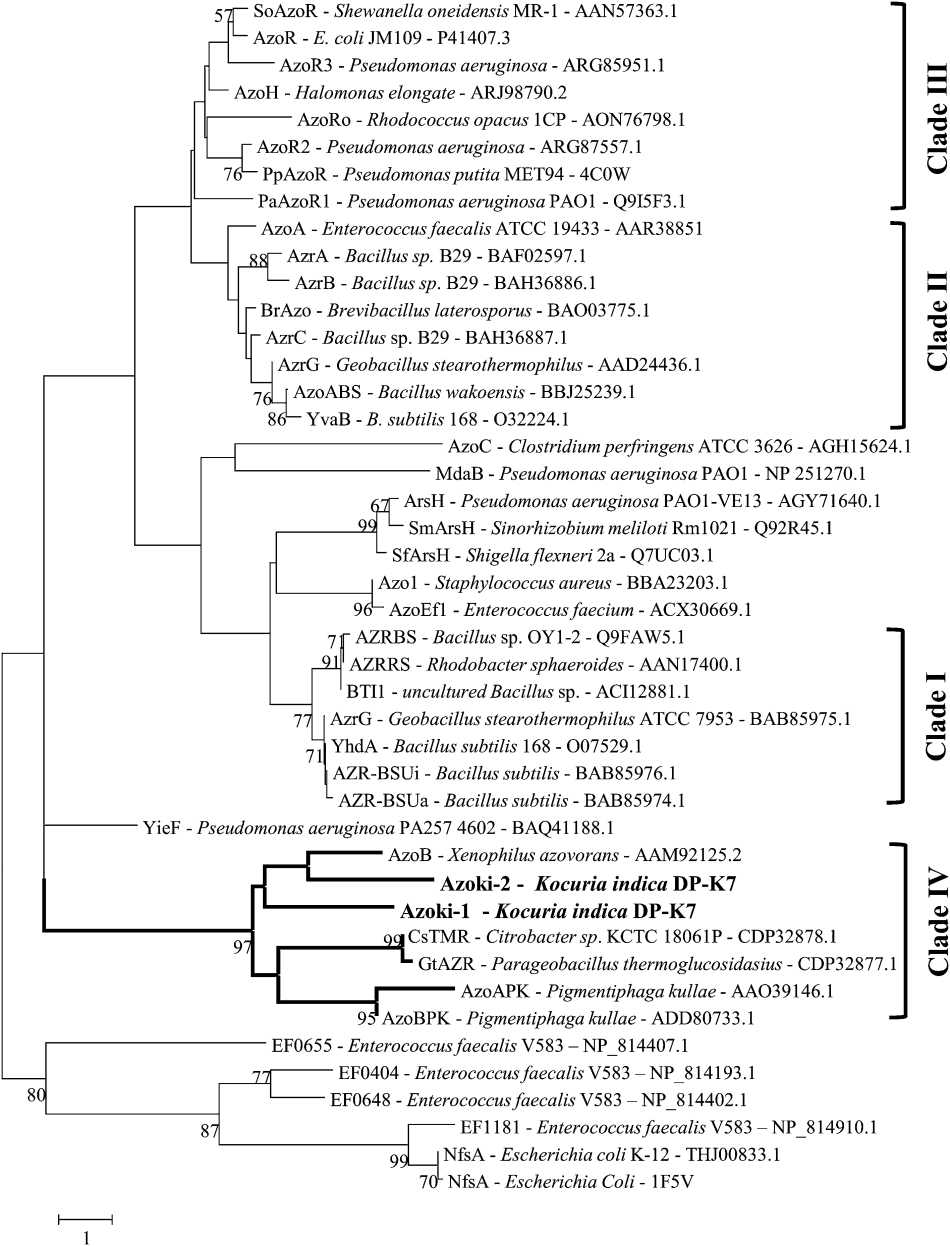
Phylogenetic tree of azoreductases. The minimum-evolution tree based on primary amino acid sequence of characterized azoreductase depicting the phylogenetic position of AzoKi-1 and AzoKi-2 from the strain DP-K7. Amino acid sequence of nitroreductases (NP_814407.1, NP_814193.1, NP_814402.1, NP_814910.1, THJ00833.1, 1F5V) were used as outgroup. Clade I, II, and III represents the members of flavin-dependent azoreductases and clade IV represents the members of flavin-independent azoreductases. The parent clade and the subclades of flavin-independent azoreductase were highlighted. Azoreductases (AzoKi-1 and AzoKi-2) from the strain DP-K7 were given in bold. Enzyme name, respective bacterial species, and accession numbers were given. Numbers at nodes indicate the percentage of bootstrap support based on 1000 bootstrap replicates. Bootstrap values below 50% were not shown. Bar 1% substitution per site

**Fig. 6:**
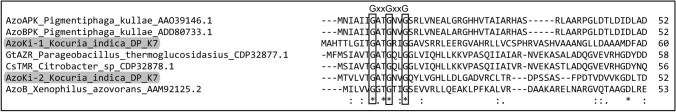
Partial sequence alignment of flavin-independent azoreductase. The amino acid sequence of azoreductases from strain DP-K7 (AzoKi-1 and AzoKi-2) aligned against previously described flavin-independent azoreductases. Enzyme name, bacterial species, and accession of respective amino acid sequence are provided. The study sequences from the strain DP-K7 are highlighted. The conserved motif GxxGxxG for NAD(P)H binding in flavin-independent azoreductase is shown in boxes

To identify potential azoreductases, sequence comparison studies were performed including flavin-independent (typically around 280–290 amino acids in length) and flavin-dependent (typically around 174–214 amino acids in length) azoreductase. The blastp of AzoB from *X. azovorans* KF46F (flavin-independent representative) against the annotated genome revealed the presence of two azoreductase coding genes *azoKi-1* (locus tag = GKZ75_04725) and *azoKi-2* (locus tag = GKZ75_12075) with identity of 28% and 27%, query cover of 95% and 98% with *E* value of 2e^−20^ and 8e^−23^, respectively. The blastp of AzoKi-1 and AzoKi-2 against other flavin-independent azoreductases namely GtAZR—*Parageobacillus thermoglucosidasius* (identity of 30% and 26.24%, query cover of 93% and 82%, *E* value of 4e^−29^ and 1e^−12^, respectively) and CsTMR—*Citrobacter* sp. (identity of 29.29% and 29.39%, query cover of 94% and 79%, E value of 3e^−28^ and 2e^−15^, respectively) showed considerable sequence similarity. The results of blastp analysis using AzoRo from *R. opacus* 1CP (flavin-dependent representative) were showing a lower similarity and thus not used. Both azoreductase coding genes *azoKi-1* and *azoKi-2* encode for 285 and 277 amino acids, respectively. Thus AzoKi-1 and Azoki-2 are larger in size compared to typical flavin-dependent azoreductase. The study of Suzuki (2019) on the diversification of bacterial azoreductase revealed that the azoreductase are classified into four different clades (clades I–IV) based on the primary amino acid sequence. The members of clades I, II, and III are typically flavin-dependent azoreductases whereas all the characterized flavin-independent azoreductases fall into clade IV. In addition, the study suggests that the enzymes of clade I comprise 174–179 amino acids while clades II and III have a length of around 198–214 amino acids. On the other hand, members of clade IV showed a wide range in length of amino acid sequence. AzoA and AzoB—*Pigmentiphaga kullae* are 200 and 203, respectively, GAZR—*Parageobacillus thermoglucosidasius* is 289, CsTMR—*Citrobacter* sp. is 287 and AzoB—*X. azovorans* is 286 amino acids (Blümel and Stolz 2003; Chen et al. 2010; Gao et al. 2015; Blümel et al. 2002). The GxGxxG NAD(P)H-binding motif was observed only in clade I members, whereas an alternate GxxGxxG motif was observed in clade IV members. Previously, Blümel et al. (2002) and Chen et al. (2010) reported that GxxGxxG NAD(P)H-binding motifs are conserved at N-terminus of flavin-independent azoreductase whereas GxGxxG NAD(P)H-binding motif of flavin-dependent azoreductase from *Bacillus* sp. strain OY1-2 was found at 106–111 position on the amino acid sequence. The reconstruction of the phylogenetic tree based on the primary amino acid sequence of characterized azoreductase showed AzoKi-1 and AzoKi-2 fall into clade IV members of azoreductases (Fig. 5). The multiple sequence alignment of AzoKi-1 and 2 (Fig. 6) with characterized flavin-independent azoreductase confirmed that the proposed NAD(P)H-binding motif (GxxGxxG) is conserved at N-terminus as observed in all the members of Clade IV azoreductase (AzoAPK—*Pigmentiphaga kullae*, AzoBPK—*Pigmentiphaga kullae*, GAZR—*Parageobacillus thermoglucosidasius*, CsTMR—*Citrobacter* sp, AzoB—*X. azovorans*). Based on the phylogenetic tree analysis, the presence of proposed GxxGxxG NAD(P)H-binding motif, multiple sequence alignment showing the position of the motif at N-terminus of protein and the primary amino acid length (285 and 277), we propose AzoKi-1 and AzoKi-2 to be flavin-independent azoreductases. In addition to azoreductase, the genome of strain DP-K7 contains genes coding for enzymes such as polyphenol oxidase, superoxide dismutase (SodC and SodA), catalase (KatA and KatE), and multifunctional dye peroxide DyP2 (Dyp2) which were also reported for dye degradation (Sarkar et al. 2017).

## Conclusion

The present study explicates the methyl red (azo dye)-degrading soil isolate *K. indica* DP-K7. The bacterial genome analysis provided some hints to withstand experimental data for biodegradation of azo dye and led to the confirmation of the presence of various azo dye-degrading enzymes including two azoreductases, a multifunctional dye peroxide DyP2, superoxide dismutase, and others. The genome showed the presence of 2754 protein-coding genes including 1997 functional assignment proteins with 598 mapping to KEGG pathway. The genome analysis, 16S rRNA gene sequencing, experimental data including RP-HPLC and the presence of azoreductase in the genome depicts that the strain DP-K7 belonging to the species *K. indica* is a potential azo dye degrader. Nevertheless, further investigation into the genome and enzymatic characterization would reveal the pathways associated with azo dye degradation by the bacterial strain DP-K7.

## Electronic supplementary material

Below is the link to the electronic supplementary material.

Supplementary file1 (XLSX 11 kb)
